# PD-1 Blockade on Tumor Microenvironment-Resident ILC2s Promotes TNF-α Production and Restricts Progression of Metastatic Melanoma

**DOI:** 10.3389/fimmu.2021.733136

**Published:** 2021-08-31

**Authors:** Emily Howard, Benjamin P. Hurrell, Doumet Georges Helou, Christine Quach, Jacob D. Painter, Pedram Shafiei-Jahani, Marshall Fung, Parkash S. Gill, Pejman Soroosh, Arlene H. Sharpe, Omid Akbari

**Affiliations:** ^1^Department of Molecular Microbiology and Immunology, Keck School of Medicine, University of Southern California, Los Angeles, CA, United States; ^2^Department of Medicine, Norris Cancer center, Keck School of Medicine, University of Southern California, Los Angeles, CA, United States; ^3^Immunometabolism, Janssen Research and Development, San Diego, CA, United States; ^4^Department of Immunology, Harvard Medical School, Boston, MA, United States

**Keywords:** innate lymphoid cell 2, cancer, melanoma, PD-1, TNF - α

## Abstract

While pulmonary ILC2s represent one of the major tissue-resident innate lymphoid cell populations at steady state and are key drivers of cytokine secretion in their occupational niche, their role in pulmonary cancer progression remains unclear. As the programmed cell death protein-1 (PD-1) plays a major role in cancer immunotherapy and immunoregulatory properties, here we investigate the specific effect of PD-1 inhibition on ILC2s during pulmonary B16 melanoma cancer metastasis. We demonstrate that PD-1 inhibition on ILC2s suppresses B16 tumor growth. Further, PD-1 inhibition upregulates pulmonary ILC2-derived TNF-α production, a cytotoxic cytokine that directly induces cell death in B16 cells, independent of adaptive immunity. Together, these results highlight the importance of ILC2s and their anti-tumor role in pulmonary B16 cancer progression during PD-1 inhibitory immunotherapy.

## Introduction

Melanoma is a highly aggressive form of cancer that spreads from primary skin sites to various organs throughout the human body (1, 2). While effective treatment is attainable if diagnosed at the early stages, 20% of melanomas diagnosed in advanced stages resist treatment (3, 4). Pulmonary metastasis is the most common progression in the late stages of cancer diagnosis (5). According to the World Health Organization, over 106,000 melanomas were diagnosed, and 7,000 new fatalities occurred in the United States in 2020.

While the rates of melanoma diagnosis continue to be alarming, molecularly targeted approaches, including inhibition of immune checkpoints such as programmed death-1 receptor (PD-1) have significantly improved patient outcomes over the last decade. Nivolumab, a Food and Drug Administration-approved antibody directed at PD-1 inhibition enhanced median overall survival in patients with metastatic melanoma (6). PD-1 binds to ligands PD-L1 or PD-L2 to act as an inhibitory immune checkpoint, negatively regulating immune cell activation and effector function (5, 7, 8). Under healthy conditions, PD-1 and its ligands ensure immune cell function homeostasis by restricting unchecked pro-inflammation in the body. In a tumor microenvironment, however, cancer cells exploit the negative regulators to elude healthy antitumor immunity (9).

Group 2 Innate Lymphoid cells (ILC2s) are tissue resident non-B and non-T innate immune cells that reside in mucosal tissues, including the lung, gut and skin (10, 11). Though primarily activated by epithelial alarmin cytokines, including interleukin (IL-) 33, IL-25 and Thymic Stromal Lymphopoietin (TSLP), to produce their effector function cytokines IL-5 and IL-13, our group and others have well documented the immense plasticity in both ILC2 stimuli and responding cytokine production and secretion (12–16). Due to their unique occupational niche in the mucosal tissue, ILC2s are primed to be first responders in the initiation of infiltrating cancer tumor cells. Recently, our group and others reported that ILC2s facultatively express PD-1 and PD-L1 when activated with alarmin cytokine IL-33 (8, 17, 18). And indeed consistent with T, NK and B cells, engagement of PD-1 on ILC2s dramatically downregulates proliferation, viability and effector function (19–21). Conversely, inhibition of PD-1 engagement results in increases in total ILC2 number, increased production of cytokines, and overall cell function (8, 17, 18). While their role in Th-2 inflammatory diseases has been well established, ILC2s’ contribution in cancer has long been controversial and requires further investigation.

Tumor Necrosis Factor alpha (TNF-α) is a cytokine that plays a vital role in melanoma tumor control in both mouse and lung cancer. Traditionally attributed to macrophages, several groups have shown that TNF-α therapy can be used clinically in combination with established therapies to enhance cancer treatment (22–24). For example, TNF-α therapy can be used to target tumor vasculature, ensuring the melanoma cells are more susceptible to anti-tumor anti-PD-1 therapy (22, 23). Moreover, T-cell derived TNF-α has been demonstrated to kill tumor cells and loss of TNF-α increases melanoma tumor cell invasion in the lung (25). It was later detailed that TNF-α kills tumor cells through enhancement of tumor oxidative stress in melanoma cells (26).

In this study, we show that B16 melanoma significantly enhances PD-1 expression on pulmonary ILC2s, effectively promoting tumor progression and limiting anti-tumor immune response. Blocking PD-1 on ILC2s however inhibits tumor growth progression and surprisingly, induces the production of ILC2-derived TNF-α. Through targeted adoptive transfer and *in vitro* studies, we identify that lack of PD-1 on ILC2s directly affects B16 tumor cell apoptosis, mediated through the cytotoxic properties of ILC2-derived TNF-α. Together, our findings give further insight into the anti-tumoral immune biology of pulmonary ILC2s and their direct role in conventional immune checkpoint therapies. Specifically, our studies can make significant progress and advance our understanding of the underlying therapeutic value of ILC2s relating to clinical cancer treatments.

## Methods

### Mouse Experiments

Experimental protocols were approved by the USC institutional Animal Care and Use Committee (IACUC) and conducted in accordance with the USC Department of Animal Resources’ guidelines. 5-10 week old age and sex matched mice were used in the studies. BALB/cByJ, RAG2^-/-^ (C.B6(Cg)-Rag2^tm1.1Cgn^/J) and *Rag2* ^-/-^ γc^-/-^(C;129S4-Rag2tm1.1Flv Il2rgtm1.1Flv/J) mice were bred in our animal facility at the Keck School of Medicine, University of Southern California (USC) (all the three strains are in BALB/c background). PD-1-deficient (*Pdcd1* ^-/-^) BALB/c mice were generated in the Sharpe laboratory as previously described (27). PD-1^-/-^ BALB/c mice were backcrossed to RAG2^-/-^ mice to create Rag2^-/-^ PD-1^-/-^ mice.

### Cell Culture

B16 cells (graciously given by Dr. Weiming Yuan) were cultured in Dulbecco’s modified eagle medium (DMEM, Sigma-Aldrich Co.) supplemented with 10% heat-inactivated fetal calf serum and a penicillin/streptomycin cocktail. Cells were routinely cultured in a humidified atmosphere of 5% Co2 at 37°C in DMEM.

### *In Vivo* Experiments and Tissue Preparation

B16 cells (2.5 × 10^5^ cells) were grown and injected by tail intravenously (i.v.) into recipient mice in a volume of 100 µL phosphate-buffered saline (PBS). Where indicated, mice were intraperitoneally (i.p.) injected with anti-asialo GM1 or isotype (Wako) every third day (day 0, 3, 6, 9, 12), and/or PD-1 blocking antibody or isotype (500µg/mouse, BioXCell) every fourth day (day 4, 8, 12). After 14 days, mice were euthanized and numbers of tumor colonies on lung surfaces were counted per field. Lungs were then collected and processed for the indicated readout as previously described (28). Briefly, the lungs were perfused with PBS and digested in Collagenase IV (MP Biomedicals, LLC) for 1 hour at 37°C. Samples were then stained and ILC2s were isolated based on the absence of common lineage markers (CD3, CD5, CD4, TCRβ, CD45R, Gr-1, CD56, CD11c, CD11b, Ter119, FcϵRIα, CD335), and the expression of CD45, ST2 and CD127.

### Human ILC2 Isolation and *In Vitro* Culture

Experimental protocols were approved by the USC Institutional Review Board (IRB) and conducted in accordance with the principles of the Declaration of Helsinki. Human blood ILC2s were isolated from total peripheral blood mononuclear cells (PBMCs) to a purity of > 95% on a FACSARIA III system as described previously (29). Briefly, human fresh blood was first diluted 1:1 in PBS 1X and transferred to SepMateTM-50 separation tubes (STEMCELL Technologies) filled with 12mL Lymphoprep™. Samples were centrifuged for 10 minutes and PBMCs were collected. CRTH2^+^ cells were then isolated using the CRTH2 MicroBead Kit, used according to the manufacturer’s conditions. Samples were then stained and ILC2s were isolated based on the absence of common lineage markers (CD3, CD5, CD14, CD16, CD19, CD20, CD56, CD235a, CD1a, CD123), and the expression of CD45, CRTH2 and CD127. Isolated ILC2s were cultured at 37°C (5x10^4^/mL) with rhIL-2 (10ng/mL) and rhIL-7 (10ng/mL) in complete RPMI (cRPMI). To make cRPMI, RPMI (Gibco) was supplemented with 10% heat-inactivated FBS (Omega Scientific), 100 units/mL penicillin and 100mg/mL streptomycin (GenClone). When indicated, human ILC2s were activated with 50ng/mL rhIL-33 for the indicated times. Human PD-1 blocking antibody or isotype was included in the culture (10µg/ml, BioXCell).

### Flow Cytometry

The following murine antibodies were used: biotinylated anti-mouse lineage CD3ϵ (145-2C11), CD4 (GK1.5), CD5 (53-7.3), TCRβ (H57-597), CD45R (RA3-6B2), Gr-1 (RB6-8C5), CD11c (N418), CD11b (M1/70), Ter119 (TER-119), FcϵRIα (MAR-1), CD335 (29A1.4), Streptavidin-FITC, PE-Cy7 anti-mouse CD127 (A7R34), APCCy7 anti-mouse CD45 (30-F11), APC anti-mouse CD49b (DX5), PE anti-mouse PD-1 (29F.1A12) were purchased from BioLegend. PerCP-eFluor710 anti-mouse ST2 (RMST2-2) was purchased from Thermofisher. Intranuclear staining was performed using the Foxp3 Transcription Factor Staining Kit (Thermofisher) per the manufacturer’s instructions. PE anti-human/mouse RelA NFκB p65 (IC_50_78P) and Alexa Fluor 647 anti-human/mouse NFκB p52 (C-5) from Biolegend and Santa Cruz Biotechnology respectively were used. Intracellular staining was performed using the BD Biosciences Cytofix/Cytoperm kit. When indicated, cells were stimulated *in vitro* for 4 hours with 50µg/mL PMA, 500µg/mL ionomycin (both Sigma) and 1µg/mL Golgi plug (BD Biosciences) before cytokine assessment. BV510 anti-mouse TNF-α (MP6-XT22, Biolegend) was used. PE-Cy7 Annexin V and DAPI were used according to the manufacturer’s instructions. The following human antibodies were used: FITC anti-human lineage cocktail including CD3 (UCHT1), CD14 (HCD14), CD16 (3G8), CD19 (HIB19), CD20 (2H7), CD56 (HCD56). Additional lineage markers were added: FITC anti-human CD235a (HI264), FITC anti-human FCϵRIα (AER-37), FITC anti-human CD1a (HI149), FITC anti-human CD123 (6H6) and FITC anti-human CD5 (L17F12). APCCy7 anti-human CD45 (HI30), PECy7 anti-human CD127 (A019D5). Live/dead fixable violet cell stain kit was used to exclude dead cells (Thermofisher) and CountBright absolute counting beads (Thermofisher) to calculate absolute cell numbers when indicated. Stained cells were analyzed on FACSCanto II and/or FACSARIA III systems and the data was analyzed with FlowJo version 10 software.

### Murine ILC2 and *In Vitro* Culture

Murine ILC2s were FACS-sorted to a purity of >95% on a FACSARIA III gating system. Isolated ILC2s were cultured at 37°C (5x10^4^/mL) for 48 hours as indicated with rmIL-2 (10ng/mL), rmIL-7 (10ng/mL), +/- rmIL-33 (10ng/ml) purchased from BioLegend in cRPMI. In the indicated experiments, cells were cultured for 48 hours with mouse PD-1 blocking antibody (10µg/ml) or appropriate isotype purchased from BioXCell. In the experiments involving Transwell plates, B16 cells (10 x 10^3^) were cultured in the bottom well of a 0.4 µm pore Transwell plate (Corning) with anti-TNF-α or isotype (BioXCell, 10µg/ml). Sorted activated PD-1^-/-^ or WT ILC2s (80 x 10^3^) were cultured in the insert as described above for 48 hours.

### Cytokine Measurements

The amounts of cytokines in culture supernatants were measured by Legendplex or ELISA MAX deluxe kit according to the manufacturer’s instructions (Biolegend).

### Adoptive Transfer

Experiments were performed as described previously (16). Mice were first inoculated with B16 cells as described above. Briefly, ILC2 from IL-33 treated PD-1^-/-^ or WT mice were sorted from the lungs. 5.0 x 10^4^ of the appropriate population of cells were transferred in PBS to Rag^-/-^γc^-/-^ mice by i.v. injection every sixth day (day 0, 6, 12). Mice were euthanized on day 14 and lung tumor burden was assessed and quantified as previously described. Lungs were then collected and processed for the indicated readout.

### Statistical Analysis

Experiments were repeated at least three times (n=4-8 each). A student t-test was used for comparisons between each group using Prism Software (GraphPad Software Inc.). Mean, Standard Deviation and degree of significance was indicated as: *p<0.05, **p<0.01, ***p<0.001.

## Results

### B16 Melanoma Induces PD-1 Expression on Pulmonary ILC2s

**Figure 1:**
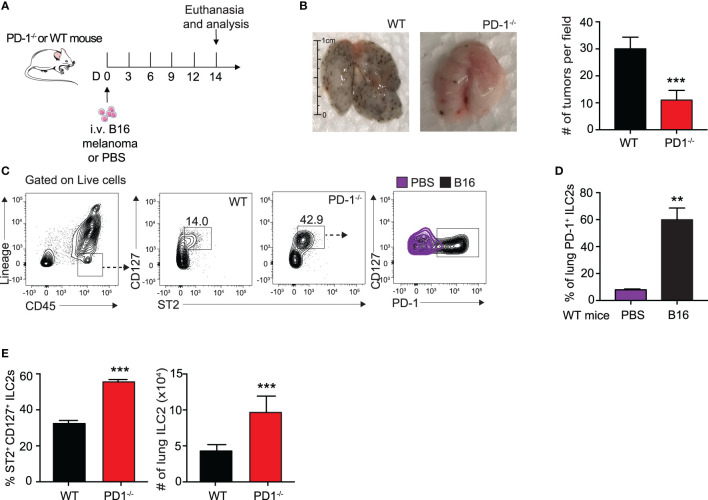
B16 melanoma induces PD-1 expression on pulmonary ILC2s. **(A)** A cohort of PD-1^-/-^ or WT mice was given an intravenous injection of B16 melanoma cells (2.5 x 10^5^) on day 0. On day 14 tumor cell colonies on the lung surface were counted per field **(B)** and presented with corresponding quantification. **(C)** Lungs were then digested and ILC2s were identified and quantified. Representative gating strategy identifying lung ILC2s and PD-1 expression by flow cytometry. **(D)** Percentage of ILC2s expressing PD-1 in lungs of WT BALB/cByJ mice intravenously injected with B16 melanoma or PBS **(E)** Percentage and total number of ILC2s in the lungs of PD-1^-/-^ or WT mice with B16 melanoma. Error bars are the mean ± SEM. Data are representative of 3 individual experiments with n=5. Student’s t-test, **p < 0.01, ***p < 0.001.

We first assessed the effect of B16 melanoma tumors on ILC2 PD-1 expression. To do this, we intravenously (i.v.) injected B16 melanoma cells or PBS control into a cohort of PD-1^-/-^ or WT mice on day 0 (**Figure 1A**). On day 14, mice were euthanized and total pulmonary tumor colonies on the surface of the lungs was assessed and quantified (**Figure 1B**). As shown in **Figure 1B**, WT mice inoculated with B16 cells displayed significantly more tumor colonies per field when compared to mice deficient in PD-1, suggesting PD-1 dramatically promotes B16 melanoma metastasis. The lungs of the mice were then digested and ILC2 PD-1 expression of WT mice with and without B16 melanoma was assessed (**Figure 1C**). The percentage of PD-1^+^ ILC2s significantly increased after B16 inoculation, from a mean of 15% PD-1^+^ ILC2s to 60% PD-1^+^ ILC2s, demonstrating B16 melanoma induces PD-1 expression on pulmonary ILC2s and may subsequently alter anti-tumor effector function (**Figure 1D**). PD-1^-/-^ mice also displayed a significant increase in the percentage and number of pulmonary ILC2s when compared to WT mice (**Figure 1E**). Together, our results suggest B16 melanoma upregulates ILC2 PD-1 expression, driving the progression of tumor growth.

### PD-1 Expression on Pulmonary ILC2s Promotes B16 Tumor Growth and Drives Melanoma-Induced Fatality

**Figure 2:**
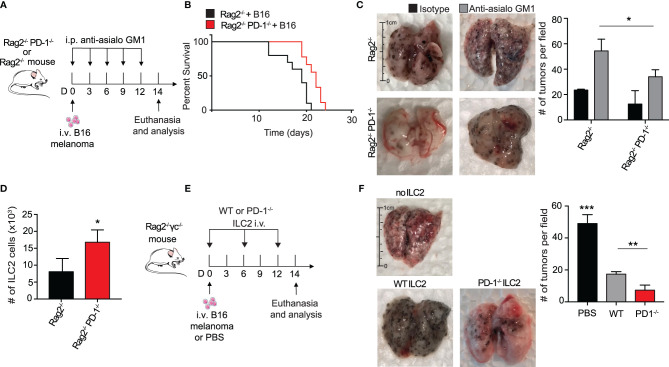
PD-1 expression on pulmonary ILC2s promotes B16 tumor growth and drives melanoma-induced fatality. **(A)** A cohort of Rag2^-/-^ or Rag2^-/-^ PD-1^-/-^ mice were intravenously injected with B16 melanoma (2.5 x 10^5^) cells on day 0. Mice were also intraperitoneally injected with anti-asialo GM1 or isotype every three days. On day 14, mice were euthanized and lung tumor burden per field was assessed and quantified. **(B)** Kaplan-Meier survival curves of Rag2^-/-^ or Rag2^-/-^ PD-1^-/-^ mice intravenously injected with B16 melanoma. **(C)** Whole lungs were isolated on day 14 and total tumors per field on lung surfaces were quantified. Representative lungs for the cohorts are presented. **(D)** Total number of lung ILC2 cells in Rag2^-/-^ or Rag2^-/-^ PD-1^-/-^ mice on day 14. **(E)** A cohort of Rag2^-/-^ γc^-/-^ mice were intravenously injected with B16 melanoma cells (2.5 x 10^5^) on day 0. Activated PD-1^-/-^ or WT ILC2s were adoptively transferred intravenously on days 0, 6 and 12. Mice were euthanized on day 14 and lung tumor burden was quantified **(F)**. Error bars are the mean ± SEM. Data are representative of 3 individual experiments with n=5. Student’s t-test, *p < 0.05, **p < 0.01, ***p < 0.001.

Several groups have reported an anti-tumor role for ILC2s in the suppression of cancers through a CD8+ T cell dependent mechanism (7, 30). To explore whether ILC2s in our context relied upon adaptive immunity for potential anti-tumor properties and cytokine production, we utilized Rag2^-/-^ PD-1^-/-^ and control Rag2^-/-^ mice. These mice lack all B and T cells, allowing us to explore more specifically the behavior of ILC2s in this microenvironment. We first gave a cohort of Rag2^-/-^ and Rag2^-/-^ PD-1^-/-^ mice i.v. injection of B16 melanoma cells on day 0 (**Figure 2A**). Since NK cells are known to have a significant anti-tumor effect on various cancers, including pulmonary melanoma, we additionally injected the mice with either anti-asialo GM1 or its isotype, an antibody used to deplete NK cells in the mouse (31). Repeated injections of anti-asialo GM1 significantly reduced the number of spleen NK cells in both Rag2^-/-^ and Rag2^-/-^ PD-1^-/-^ mice (**Supplementary 1A**). In a first cohort, we found that Rag2^-/-^ PD-1^-/-^ mice survived significantly longer as compared to the Rag2^-/-^ control mice (**Figure 2B**). Additionally, tumor metastasis drastically decreased in Rag2^-/-^ PD-1^-/-^ mice as compared to the Rag2^-/-^ control (**Figure 2C**). As a proof of concept, we demonstrated that mice injected with the isotype as opposed to the NK depleting antibody do indeed have less tumor colonies on the surface of their lungs, consistent with published results (**Figure 2C**) **(** 31). However PD-1 expression on ILC2s greatly enhances tumor progression, as evidenced by the increase in tumors in Rag2^-/-^ control mice depleted of NK cells. Importantly, we also found that the number of total ILC2 cells were significantly increased in Rag2^-/-^ PD-1^-/-^ mice, further suggesting ILC2s play an important anti-tumor role independent of surrounding B and T cells (**Figure 2D**). In order to directly test whether ILC2s had a direct effect on the inhibition of tumor progression, we injected B16 cells into a cohort of Rag2^-/-^ γc^-/-^ mice, a strain that lacks all B, T, and innate lymphoid cells (**Figure 2E**). Concurrently we adoptively transferred IL-33-activated pulmonary ILC2s FACS-sorted from either PD-1^-/-^ or WT mice every six days, using our previously established protocol (16). On day 14, lungs were removed and tumor burden on the surface was assessed (**Figure 2F**). Strikingly, mice that did not receive either ILC2 phenotype demonstrated a significant increase in tumor metastasis as compared to the mice adoptively transferred either WT or PD-1^-/-^ ILC2s, suggesting that ILC2s do indeed play an essential role in an anti-tumor immune response independent of adaptive immunity. Most importantly, however, the mice adoptively transferred with PD-1^-/-^ pulmonary ILC2s had the least number of B16 tumor colonies at day 14, indicating that PD-1 inhibits important aspects of anti-tumor ILC2 response *in vivo.* Interestingly, total number of pulmonary ILC2s was not significantly different between the adoptively transferred groups (**Supplementary 1B**), indicating the decrease in tumor burden was independent of a difference in ILC2 number. Taken together, our results suggest that PD-1 deficiency in ILC2s play a specific role in anti-tumor immunotherapy in the inhibition of tumor progression and improved survival.

### PD-1 Deficiency on IL-33 Stimulated ILC2s Enhances TNF-α Expression and Phosphorylation of Canonical NFκB Pathway

**Figure 3:**
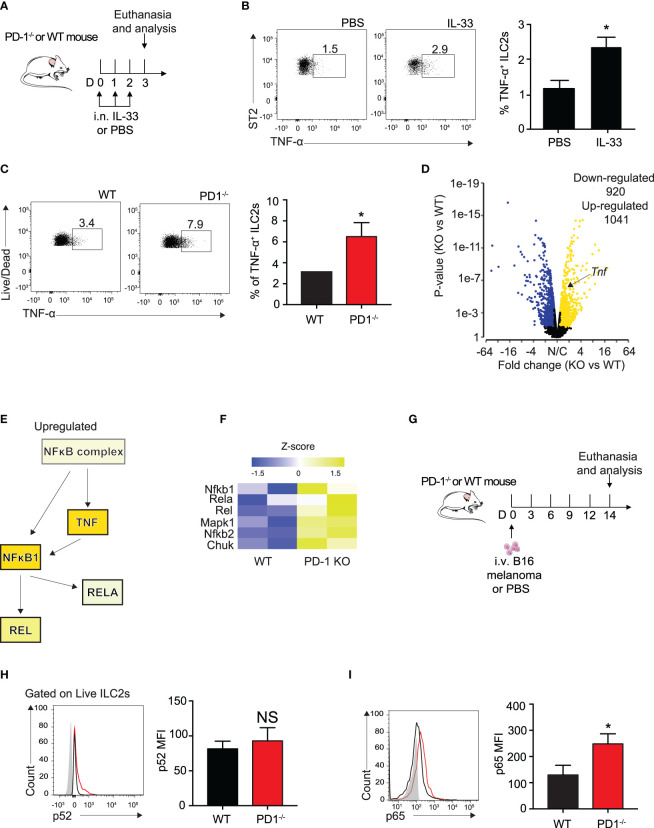
PD-1 deficiency on IL-33 stimulated ILC2s enhances TNF-a expression and phosphorylation of canonical NFκB pathway. **(A)** A cohort of WT mice were intranasally challenged with IL-33 (0.5µg) for three consecutive days, days 0-2. On day 3, mice were euthanized and percentage of TNF-α^+^ ILCs was quantified in **(B)**. **(C)** A cohort of WT or PD-1^-/-^ mice were intranasally challenged with IL-33 (0.5µg) for three consecutive days, days 0-2. On day 3, mice were euthanized and percentage of TNF-α^+^ ILCs was quantified. **(D)** Volcano plot comparison of whole transcriptome gene expression of sorted PD-1^-/-^ ILC2s and WT control, n=2. Differentially expressed genes (defined as statistically significant adjusted p-value<0.05) with changes of at least 1.45 fold-change (FC) are shown in yellow (upregulated genes) or blue (downregulated genes). **(E)** Upregulated (yellow) genes in the NFκB pathway and corresponding heatmap representation **(F). (G)** A cohort of PD-1^-/-^ or WT mice was given an intravenous injection of B16 melanoma (2.5 x 10^5^) on day 0. On day 14, lung ILC2s were identified as defined in **Figure 1C** and transcription factors involved in the NFκB pathway were measured for phosphorylation by intranuclear flow cytometry. **(H)** Representative flow cytometry plots and corresponding quantification of phosphorylation levels of transcription factor p52. **(I)** Representative flow cytometry plots and corresponding quantification of phosphorylation levels of transcription factor p65. Error bars are the mean ± SEM. Data are representative of 3 individual experiments with n=5. Student’s t-test, NS = Non-statistical, *p < 0.05.

Several groups have previously reported IL-33 to be present in the melanoma tumor microenvironment, though it is unclear whether the IL-33 is secreted from the melanoma cells themselves or the lung epithelial layer due to injury (32, 33). To further explain the relationship of PD-1 expression with the loss of important ILC2 anti-tumor properties in a more controlled setting, we intranasally (i.n.) challenged a cohort of WT mice with either rmIL-33 or PBS for three consecutive days (**Figure 3A**). After three challenges, a variety of intracellular cytokines important for the arrest of tumor growth was assessed. Surprisingly, we found a significant increase ILC2 TNF-α production, a potent anti-tumor cytokine known to severely inhibit melanoma growth amongst others (**Figure 3B**). We repeated the same experiment on a cohort of WT and PD-1^-/-^ mice and remarkably found that PD-1 deficiency combined with IL-33 stimulation further increased the percentage of TNF-α^+^ ILC2s (**Figure 3C**). In addition to TNF-α, we previously showed that Th-2 cytokines, including IL-5 and IL-13 were increased in ILC2s deficient in PD-1 (8). Interestingly, GM-CSF was also upregulated, both at an RNA sequencing level (8) and at the protein level (**Supplementary 2A**).

TNF-α production is controlled primarily by the canonical NFκB pathway (25). To elucidate the mechanism by which ILC2s produce TNF-α, we reanalyzed our group’s previously published RNA sequencing data of sorted, IL-33-activated ILC2s from PD-1^-/-^ and WT mice with the new focus of genes involved in the canonical NFκB pathway (8). Samples were characterized by group according to RNA quality and gene expression (**Supplementary 2B**) and differential gene expression between the two groups was plotted on a volcano plot (Fold change > 1.45, P<0.05, **Figure 3D**). The results remained consistent with our previous findings in that ILC2s deficient in PD-1 expression had significantly upregulated *Tnf* expression (Fold change = 1.5, P<0.001) (**Figure 3D**).

Further transcriptomic analysis by Ingenuity Pathway Analysis (IPA) suggests the NFκB complex and related genes upstream of TNF-α production are increased (**Figure 3E**). And indeed, heatmap analysis focusing on genes highlighted in **Figure 3E** are upregulated in activated PD-1^-/-^ ILC2s (**Figure 3F**). As the results from this analysis are from IL-33-activated ILC2s, we questioned whether NFκB upregulation was present in the ILC2s of our B16 melanoma model. We therefore injected B16 cells into PD-1^-/-^ and WT mice as described above (**Figure 3G**) and measured phosphorylation levels of p52 and p65, non-canonical and canonical members of the NFκB pathway respectively, in pulmonary ILC2s on day 14 (**Figures 3H, I** **)**. p52 phosphorylation levels were unchanged in our model (**Figure 3H**). In line with the findings from the IL-33-activated RNA sequencing data, phosphorylation levels of p65 were significantly inhibited in WT ILC2s isolated from the TME as compared to PD-1^-/-^ ILC2s (**Figure 3I**). Altogether, our data suggest that the B16 melanoma tumor microenvironment induces phosphorylation of the NFκB pathway in PD-1 deficient pulmonary ILC2s, potentially stimulating ILC2 TNF-α production.

### Blocking PD-1 on Tumor Microenvironment ILC2s Increases TNF-α Production and Enhances Cytotoxic Properties *Ex Vivo*

**Figure 4:**
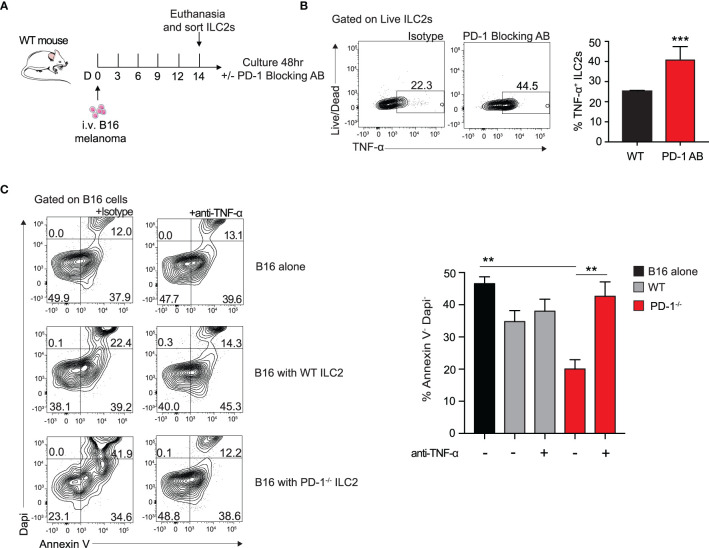
Blocking PD-1 on ILC2s increases TNF-α production and enhances cytotoxic properties. **(A)** A cohort of WT mice was given an intravenous injection of B16 melanoma on day 0. On day 14 lung ILC2s were sorted by flow cytometry and cultured with isotype or PD-1 blocking antibody for 48 hours. **(B)** After 48 hours, ILC2 cells were harvested and measured for TNF-α expression by intracellular flow cytometry. Representative flow cytometry plots and corresponding quantification are presented as percent TNF-α^+^ ILC2s. **(C)** B16 melanoma cells were cultured in the bottom well of 0.4 µm Transwell plate. PD-1^-/-^ or WT ILC2s were cultured in the top well insert at 8:1 ratio with B16 melanoma cells respectively for 48 hours. In the condition specified, anti-TNF-α or isotype (10µg/ml) was included in the bottom well of the culture. After 48 hours, B16 cells were collected and stained with apoptosis kit to assess viability. Representative flow cytometry plots and corresponding quantification are presented. Error bars are the mean ± SEM. Data are representative of 3 individual experiments with n=5. Student’s t-test, **p < 0.01, ***p < 0.001.

PD-1 blocking antibodies are currently used therapeutically to upregulate anti-tumor immune responses in human patients (9). To investigate the *in vitro* effect of PD-1 blocking antibody on TME ILC2s, we injected a cohort of WT mice with B16 melanoma on day 0 (**Figure 4A**). After 14 days, the lungs were digested and ILC2s were FACS-sorted as previously demonstrated (10). ILC2s were then cultured with recombinant mouse (rm)IL-2, IL-7, IL-33 for 48 hours, with the addition of the mouse PD-1 blocking antibody or the isotype. After 48 hours, cells cultured with the PD-1 blocking antibody demonstrated a significant upregulation of TNF-α production in the ILC2s by intracellular flow cytometry, from 22% to 45% (**Figure 4B**). Excitingly, a relatively high percentage of ILC2s cultured with either the antibody or the isotype produced TNF-α, suggesting the B16 melanoma context from which they originated primed the cells for TNF-α production. Moreover, blocking PD-1 significantly increased the percentage of TNF-α^+^ ILC2s, further suggesting a novel mechanism by which PD-1 blocking antibody may lead to tumor inhibition by ILC2s *in vivo*.

Since TNF-α is a well-established inhibitor of tumor growth in a variety of cancers, we wondered if the ILC2-derived TNF-α had any direct effect on B16 melanoma cells. To test this, we first cultured B16 melanoma cells in the bottom well of a 0.4µm Transwell plate with either a mouse neutralizing TNF-α antibody or the isotype. We next FACS-sorted activated PD-1^-/-^ or WT ILC2s to culture on top of the insert. After 48 hours, we collected the B16 cells from the bottom well and used Dapi and Annexin V to assess early apoptotic and dead cells (**Figure 4C**). Cells cultured with PD-1^-/-^ ILC2s displayed a significant decrease in viability compared to both those cultured with WT ILC2s and those cultured alone. Importantly, cells cultured with anti-TNF-α neutralizing antibody saw a significant restoration in viability, suggesting that TNF-α production from ILC2 has a direct effect on melanoma apoptosis. Together these results suggest a novel anti-tumor mechanism of action by which PD1 deficiency on ILC2s affects melanoma cells.

### Blocking ILC2 PD-1 Expression Increases TNF-α Production and Inhibits Tumor Progression *In Vivo*

**Figure 5:**
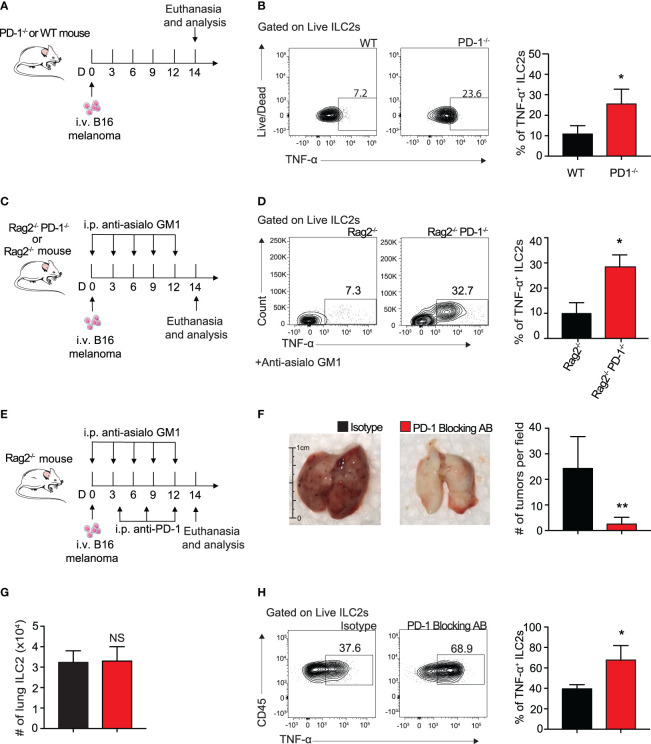
Blocking ILC2 PD-1 expression increases TNF-α production and inhibits tumor progression *in vivo*. **(A)** A cohort of PD-1^-/-^ or WT mice was given an intravenous injection of B16 melanoma cells (2.5 x 10^5^) on day 0. On day 14 lungs were digested and percentage of TNF-α^+^ ILC2s was quantified **(B)**. **(C)** A cohort of Rag2^-/-^ or Rag2^-/-^ PD-1^-/-^ mice were intravenously injected with B16 melanoma (2.5 x 10^5^) cells on day 0. Mice were also intraperitoneally injected with anti-asialo GM1 or isotype every three days. On day 14, mice were euthanized and percentage of TNF-α^+^ ILC2s was quantified **(D)**. **(E)** A cohort of Rag2^-/-^ mice were intravenously injected with B16 melanoma cells (2.5 x 10^5^) on day 0. Mice were intraperitoneally injected with anti-asialo GM1 or isotype every three days. Mice were also intraperitoneally injected with PD-1 blocking antibody or isotype (500µg/mouse) every four days. On day 14, mice were euthanized and lung tumor burden per field was assessed and quantified **(F)**. **(G)** Total number of lung ILC2 cells present on day 14. **(H)** Representative flow cytometry plots and corresponding quantification of TNF-α^+^ ILC2s present in the lung on day 14. Error bars are the mean ± SEM. Data are representative of 3 individual experiments with n=5. Student’s t-test, NS = Non-statistical, *p < 0.05, **p < 0.01.

To determine if ILC2 TNF-α production was taking place in our *in vivo* B16 melanoma models, we i.v. injected B16 melanoma to a cohort of WT and PD-1^-/-^ mice as described above (**Figure 5A**). And indeed, pulmonary ILC2s from PD-1^-/-^ statistically increased the percentage of TNF-α production 3-fold on day 14 by intracellular flow cytometry (**Figure 5B**). Of note, this was again demonstrated in our previous *in vivo* model utilizing mice deficient in adaptive immunity (**Figures 5C, D** **)**. As previously mentioned, PD-1 blocking antibodies are vital immune checkpoint inhibitors used for therapeutic means in today’s diagnostic world. To determine if the phenotypic difference exhibited in Rag2^-/-^ and Rag2^-/-^ PD-1^-/-^ mice is clinically translatable, we injected a cohort of Rag2^-/-^ mice with B16 melanoma cells (**Figure 5E**). In addition to the NK-depleting anti-asialo GM1 injections, we included repeated i.p. injections of mouse PD-1 blocking antibody or its isotype every four days. After 14 days, the lungs were removed for tumor colony assessment and quantification (**Figure 5F**). Mice that received the PD-1 blocking antibody demonstrated a significant decrease in the number of tumor colonies on the lung surface. Of note, the number of pulmonary ILC2 cells between the two groups was unchanged (**Figure 5G**). However, there remained a significant increase in the percentage of TNF-α producing ILC2s in mice that received the PD-1 blocking antibody (**Figure 5H**). Together these results suggest that the ILC2s treated with PD-1 blocking antibody have significant clinical relevance for anti-tumorigenic function.

### Blocking PD-1 Engagement on Human Blood ILC2 Cells Increases TNF-α Secretion

**Figure 6:**
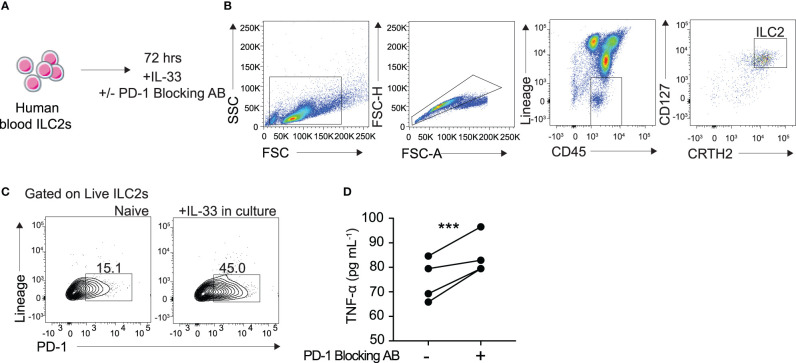
Blocking PD-1 engagement on human blood ILC2 cells increases TNF-α secretion. **(A)** ILC2s from the blood were isolated from four human donors and cultured for 72 hours in cRPMI with IL-33, survival cytokines, and PD-1 blocking antibody or isotype. **(B)** Representative flow cytometry plots demonstrating gating strategy for ILC2 sorting protocol. **(C)** Representative flow cytometry plots of PD-1 expression on ILC2s cultured with and without IL-33 after 72 hours. **(D)** Supernatant was collected after 72 hours and TNF-α secretion was measured by Luminex. Error bars are the mean ± SEM. Student’s t-test, ***p < 0.001.

To better understand the clinical relevance of PD-1 inhibition in TNF-α production, we sought to investigate its role in human blood ILC2s. Human ILC2s were identified and sorted as CD45+ lineage− CD127+ CRTH2+ (**Figures 6A, B** **)**. The cells were then cultured in cRPMI with recombinant human (rh)IL-2, IL-7, with and without IL-33, and with either human PD-1 blocking antibody or isotype for 72 hours as previously described. PD-1 expression in human ILC2s cultured with IL-33 was significantly induced after 72 hours (**Figure 6C**). Additionally, TNF-α secretion was significantly increased in supernatant of human ILC2s cultured with the PD-1 blocking antibody as compared to the isotype (**Figure 6D**). These data, together with the previous findings, provide insight into an exciting new mechanism of action by which human ILC2s can be utilized for anti-tumor therapies.

## Discussion

Overall this study introduces a novel anti-tumor role of pulmonary ILC2s in the context of metastatic melanoma through the production of TNF-α. We demonstrate that B16 melanoma significantly induces PD-1 expression on pulmonary ILC2s, thereby dampening pro-inflammatory properties vital to the inhibition of tumor progression. Blocking or deficiency of PD-1 on ILC2s slows tumor progression independent of adaptive immunity. Additionally, inhibition of PD-1 significantly enhances human and mouse ILC2 TNF-α production, a cytokine with the potential to directly induce apoptosis in B16 melanoma cancer cells.

Many groups have reported the importance of ILC2 involvement in both anti-tumor and pro-tumoral immunity in a variety of mouse and human cancers. For example, ILC2s’ rapid secretion of inflammatory cytokines IL-13 and IL-4 in human cells have been reported to promote tumor growth by recruitment of monocytic myeloid-derived suppressor cells, immune cells important for the inhibition of anti-tumor immunity (34). And indeed, in prostate cancer, Trabanelli et al. found that PGD2 secreted from tumor cells led to increased numbers of ILC2s in mouse and human prostate cancer, resulting in an immunosuppressive pathway primed for tumor progression (35). In contrast, Moral et al. published that increased number of ILC2s in human pancreatic ductal adenocarcinoma led to improved survival, relying on the cells’ ability to recruit and activate CD8+ T cells (30). In similar duality, Schuijs et al. reports that IL-33 treatment enhances ILC2 IL-5 immune response in the lung, thus activating eosinophils that subsequently suppress vital, anti-tumor Th-1 immunity, and lending to increased lung cancer metastasis and mortality (36). Lucarini et al, on the other hand, agrees that IL-33 activates eosinophils, but demonstrates their cytotoxic capabilities against lung metastatic tumors and successive growth delay (37). It is therefore evident that additional studies investigating the individual contribution of ILC2s to tumor progression are required.

The plasticity of ILC2 cytokine secretion has been well established. ILC2s are not traditionally credited with TNF-α secretion. We show that both mouse and human ILC2s stimulated *in vitro* with IL-33 do indeed produce TNF-α, and this is increased with PD-1 deficiency or inhibition. ILC2s deficient in PD-1 also exhibit an increase in Th-2 cytokines, such as IL-5 and IL-13 as previously published by our group (8). Furthermore, we also see an increase in GM-CSF at both the RNA sequencing and protein level (8). These cytokines may contribute synergistically with TNF-α in the observations reported here *in vivo*. However, our results suggest *ex vivo* neutralization of TNF-α alone is sufficient to abrogate the anti-tumor effect of PD-1 deficient ILC2s. Furthermore, in the B16 melanoma model, the percentage of TNF-α-producing ILC2s significantly increases, suggesting the melanoma tumor microenvironment is primed to allow for ILC2 TNF-α secretion. TNF-α is secreted after activation of the NFκB pathway, but exactly how PD-1 deficiency enhances production upstream remains unclear. Metryka et al. details regulation of PI3K/AKT and IKKs are important for NFκB activation and subsequent TNF-α secretion, but whether these are involved in our model remains to be investigated (38). Moreover, whether TME IL-33 alone or in combination with additional unidentified stimuli induces the high levels of ILC2 TNF-α in our melanoma model requires further exploration. Golebski et al. reported increased ILC2 derived TNF-α production after stimulation with IL-1B, IL-23 and TGF-B (13). Maggi et al. also demonstrated that human ILC2s produce TNF-α after various stimulations (39). Based on our combined results of PD-1 deficient/inhibitory mouse models and cytotoxicity assay, we propose a novel, direct role of ILC2-derived TNF-α in the apoptosis of B16 melanoma cells that is enhanced by anti-PD-1 therapy in the significant delay of melanoma tumor growth.

One limitation of our study is that the NK depleting antibody used, anti-asialo GM1, may recognize a subset of basophils *in vivo* as reported before (40). However, we believe it is unlikely that basophils play a role in our melanoma model.

In summary, this report details a previously unrecognized immunotherapeutic role for blocking PD-1 on ILC2s in B16 melanoma metastasis. The identification of potent, anti-tumor cytokine, TNF-α, dramatically induced in pulmonary ILC2s is vitally important for a cell that is uniquely positioned to act as the first responder during tumor infiltration. Based on our findings together, we propose an essential role of both human and mouse ILC2s in PD-1 immunotherapies dependent on their dramatic ability to produce TNF-α. We believe the knowledge gained by this report increases the therapeutic potential of anti-PD-1 therapies.

## Data Availability Statement

The raw data supporting the conclusions of this article will be made available by the authors, without undue reservation.

## Ethics Statement

The studies involving human participants were reviewed and approved by USC Institutional Review Board. The patients/participants provided their written informed consent to participate in this study. The animal study was reviewed and approved by USC institutional Animal Care and Use Committee.

## Author Contributions

EH designed, performed, and analyzed experiments and wrote de manuscript. BH, DH, PS-J, JP, MF, and CQ helped perform experiments and animal husbandry for experiments. OA supervised, designed the experiments, interpreted the data, and finalized the manuscript. All authors contributed to the article and approved the submitted version.

## Funding

This article was financially supported by National Institutes of Health Public Health Service grants R01 ES025786, R01 ES021801, R01 HL144790, R01 HL151493, R01 AI145813 and R01 HL151769 (OA).

## Conflict of Interest

AS declares that they have patents/pending royalties on the PD-1 pathway from Roche and Novartis. PS declares that they are an employee of Janssen R&D. OA declares that they receive grant support from the NIH.

The remaining authors declare that the research was conducted in the absence of any commercial or financial relationships that could be construed as a potential conflict of interest.

## Publisher’s Note

All claims expressed in this article are solely those of the authors and do not necessarily represent those of their affiliated organizations, or those of the publisher, the editors and the reviewers. Any product that may be evaluated in this article, or claim that may be made by its manufacturer, is not guaranteed or endorsed by the publisher.
