# Supplementary material for: PD-1 Blockade on Tumor Microenvironment-Resident ILC2s Promotes TNF-α Production and Restricts Progression of Metastatic Melanoma
Source: Front Immunol. 2021 Aug 31;12:733136. doi: 10.3389/fimmu.2021.733136 (PMC8438316; doi:10.3389/fimmu.2021.733136)
Supplement: Supplementary file 1 [file DataSheet_1.docx]

**Supplemental 1**

**
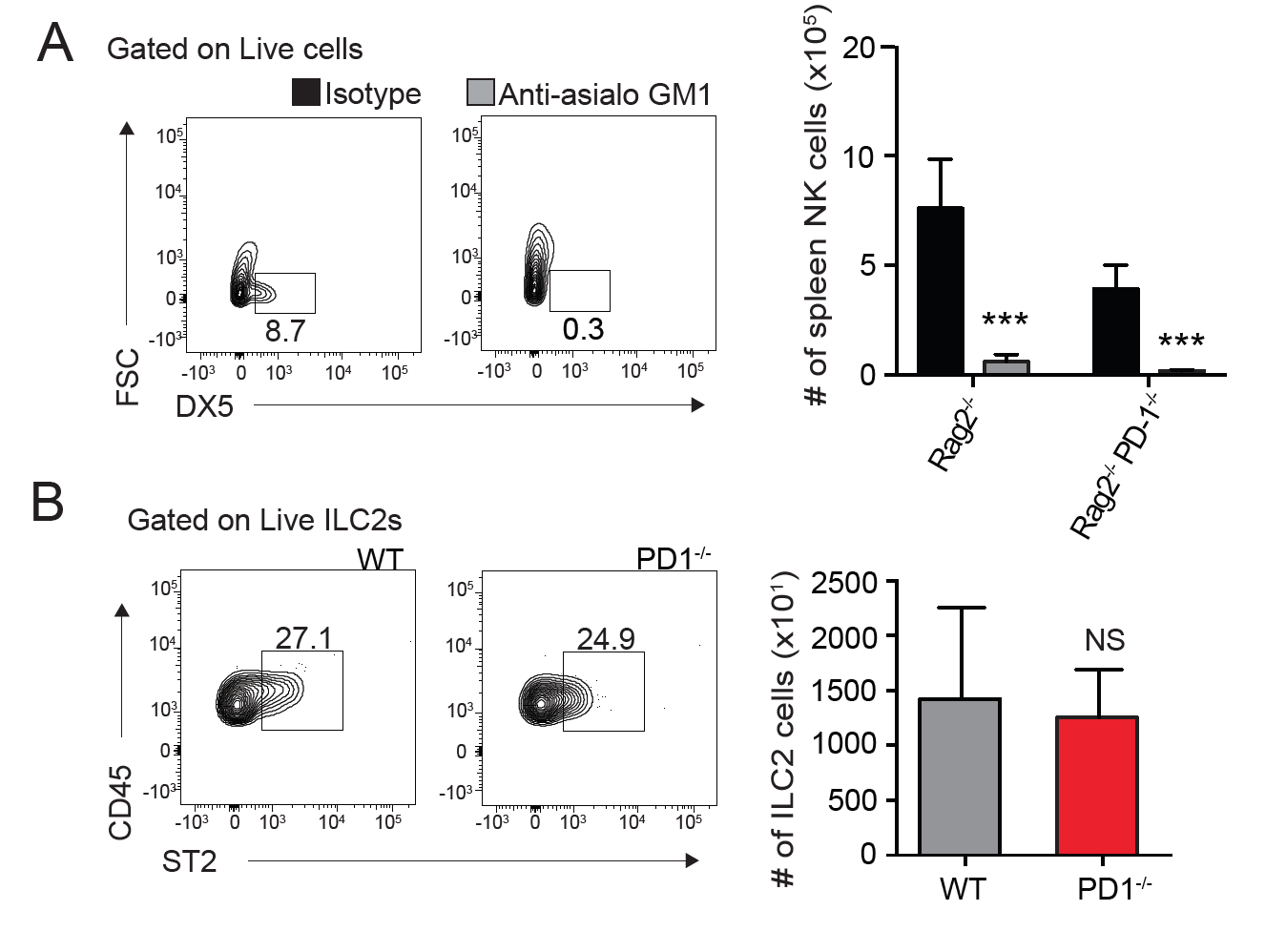
 PD-1 expression on pulmonary ILC2s promotes B16 tumor growth and drives melanoma-induced fatality.**

(**A**) Representative flow cytometry plots of NK cell depletion in the spleen of Rag2^-/-^ or Rag2^-/-^ PD-1^-/-^ mice after repeated intraperitoneal injections with anti-asialo GM1 or isotype as described in **Figure 2A**. Corresponding quantification is represented as number of total spleen NK cells.

(**B**) Representative flow cytometry plots and corresponding quantification of total number of lung ILC2 cells present on day 14.

Error bars are the mean ± SEM. Data are representative of 3 individual experiments with n=5. Student’s t-test, p < 0.05, **p < 0.01, ***p < 0.001.

**Supplemental 2**


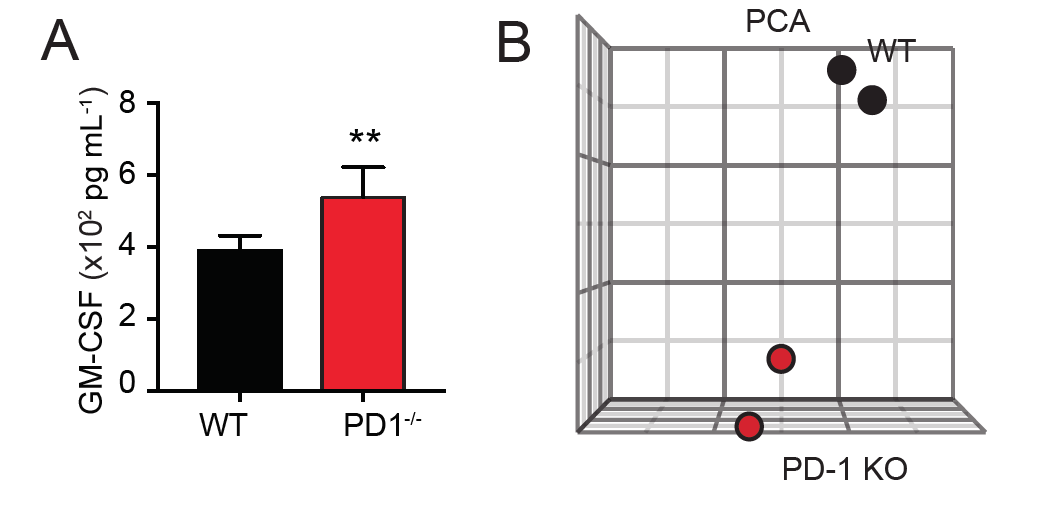


**PD-1 deficiency on IL-33 stimulated ILC2s enhances TNF-a expression and phosphorylation of canonical NFκB pathway**

(**A**) Secreted levels GM-SCF by PD-1^-/-^ and WT ILC2s cultured for 48 hours as measured by ELISA.

(**B**) Principal component analysis (PCA) of the normalized RNA seq data transcripts per million (TPM) of activated PD-1^-/-^ or WT ILC2s.
